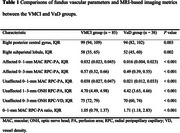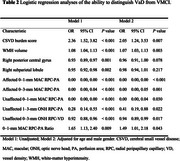# Optical Coherence Tomography Angiography for Assessing Cognitive Function in Patients with Internal Carotid Artery Stenosis

**DOI:** 10.1002/alz70856_101924

**Published:** 2025-12-25

**Authors:** Panpan Shen, Sheng Zhang, Yu Geng

**Affiliations:** ^1^ Zhejiang Chinese Medical University, Hangzhou, Zhejiang, China; ^2^ Zhejiang Provincial People's Hospital, Hangzhou, Zhejiang, China

## Abstract

**Background:**

We investigated retinal and choroidal microvascular parameters as potential biomarkers for vascular cognitive impairment (VCI) in patients with internal carotid artery stenosis (ICAS).

**Method:**

We enrolled 123 asymptomatic ICAS patients and categorized them into vascular mild cognitive impairment (VMCI) and vascular dementia (VaD) using the Montreal Cognitive Assessment. Optical coherence tomography angiography (OCTA) was used to evaluate vessel densities and perfusion areas in various retinal layers. Magnetic resonance imaging (MRI)‐based neuroimaging biomarkers for cerebral small vessel disease (CSVD) were also assessed. LASSO logistic regression identified predictor variables, and receiver operating curve (ROC) analysis assessed the ability to distinguish between VMCI and VaD.

**Result:**

The radial peripapillary capillary (RPC) perfusion area in both affected and unaffected eyes correlated positively with VaD, whereas CSVD burden score and white‐matter hyperintensity (WMH) volume correlated negatively (all *P* < 0.05). ROC analysis showed that the RPC perfusion area of the affected eye had superior discriminatory power for distinguishing VaD from VMCI compared with CSVD burden score (Z = 1.99, *p* = 0.047) and WMH (Z = 1.97, *P* = 0.049). The optimal cutoff value for the 0–1‐mm macular RPC perfusion area was determined to be 0.068 mm^2^.

**Conclusion:**

OCTA‐derived RPC perfusion area effectively differentiates VaD from VMCI, suggesting its potential as a noninvasive diagnostic method to support clinical decision‐making and prevent cognitive decline in ICAS patients.